# *Pitcairnia* L’Hér (Bromeliaceae-Pitcairnioideae) diversity and distribution in the Brazilian Amazon

**DOI:** 10.3897/BDJ.12.e136458

**Published:** 2024-12-12

**Authors:** Brenda de Moura Carvalho, Deisy Pereira Saraiva, Thuane Bochorny, Rafaela Campostrini Forzza

**Affiliations:** 1 Jardim Botânico do Rio de Janeiro, Rio de Janeiro, Brazil Jardim Botânico do Rio de Janeiro Rio de Janeiro Brazil; 2 Universidade Federal do Amazonas, Manaus, Brazil Universidade Federal do Amazonas Manaus Brazil; 3 Instituto Chico Mendes de Conservação da Biodiversidade, Prado, Brazil Instituto Chico Mendes de Conservação da Biodiversidade Prado Brazil

**Keywords:** biodiversity, bromeliads, conservation, geographical patterns, river influence, taxonomy, endemic species, species richness

## Abstract

**Background:**

The Amazon Rainforest, a paramount source of global biological diversity, faces challenges due to its understudied species richness, an insufficient investment in research and escalating rates of deforestation. Thus, acquiring additional data, especially for species distributions is crucial to fill knowledge gaps and guide forthcoming research and conservation initiatives in areas that have been inadequately sampled. This study contributes to addressing these challenges by offering new insights into the diversity and distribution of *Pitcairnia* species in the Brazilian Amazon.

**New information:**

We recorded 24 species of *Pitcairnia* for the Brazilian Amazon, of which nine species are endemic to Brazil and seven are endemic to the Brazilian Amazon Basin. Most species can be rupicolous and have distributions in the Amazon Basin that are influenced by rivers and other watercourses. One species (*P.aureobrunnea* Rauh) was recorded for the first time in the Brazilian Amazon and two new *Pitcairnia* species were discovered and are being described in separate articles, contributing to the expanding body of scientific knowledge.

## Introduction

The Amazon Rainforest stands out for its vast extension and is the world’s most important source of biological diversity (Cardoso et al. 2017, Antonelli et al. 2018). Brazil has more than half of the Amazon Region (MMA – Ministério do Meio Ambiente e Mudança do Clima 2004), which covers 58.9% of the country and is mainly in the North Region (IBGE – Instituto Brasileiro de Geografia e Estatística 2022). Despite having the highest estimates of species richness (Raedig et al. 2010, ter Steege et al. 2016), these figures may still underestimate the actual diversity, as a significant portion of the flora remains poorly studied (Hopkins 2019). This knowledge gap is attributed to a lack of investments in basic research in the region (Cardoso et al. 2017, Hopkins 2019), causing the region to be severely undersampled (Stropp et al. 2020, Gomes‐da‐Silva and Forzza 2021). Further, an increase in deforestation rates (INPE – Instituto Nacional de Pesquisas Espaciais 2020) continues to threaten diversity across the Amazon Basin (Hopkins 2019, Stropp et al. 2020).

*Pitcairnia* L’Hér. is one of the largest genera of Bromeliaceae, comprising about 412 species (Gouda et al. 2024, POWO 2024). It is the most widely distributed genus of Pitcairnioideae, occurring in Mexico, Central America, the Antilles and South America, with the highest species richness in the Andean Region and one disjunct species, *Pitcairniafeliciana* (A. Chev.) Harms & Mildbr., in Guinea, West Africa (Smith and Downs 1974, Martinelli and Forzza 2006, Moura et al. 2019). In Brazil, the genus is represented by 55 species, of which 41 are endemic (Saraiva et al. 2024). These species have distinct and consistent distribution patterns, being restricted to specific phytogeographic domains based on their habitat preferences, primarily occurring on rocky outcrops in the Atlantic Forest, in campos rupestres (i.e. rocky outcrops) in the Cerrado and on rocky outcrops and near rivers in the Amazon Rainforest (Saraiva et al. 2015, Saraiva et al. 2024).

The Bromeliaceae species in the Amazon Basin, including those of *Pitcairnia*, have been insufficiently collected and are under-represented in herbarium collections (Sousa and Wanderley 2007, Saraiva et al. 2024). Thus, additional data, especially on the distribution of species, are indispensable to fill knowledge gaps and guide future research and conservation efforts in undersampled areas (BFG [The Brazil Flora Group] 2021). In this study, we provide and discuss information regarding the aspects of geographical ranges of *Pitcairnia* species in the Brazilian Amazon with the goal of contributing to filling these significant knowledge gaps.

## Sampling methods

### Sampling description

To analyse the distribution ranges of the *Pitcairnia* species, a dataset of geographic distributions, based on specimens of all sampled species, was manually compiled in Microsoft Excel using herbarium records and online data available through the biodiversity portals Jabot (2024), Reflora (2024) and speciesLink (2024). Collections deposited in the BHCB, CEN, ESA, HB, HERBAM, HSTM, HUAM, HURB, IAN, IBGE, INPA, K, MBM, MG, MO, NY, R, RB, RO, RON, UFACPZ, US and V Herbaria (Thiers 2024) were consulted either in person or online.

Distribution points were carefully curated and only specimens identified or reviewed by taxonomists of the group were considered. Duplicates were excluded and only specimens analysed in person or through digitised images were included. Specimens without original coordinates were not added to avoid errors in location. For accuracy, locality data from specimen labels were cleaned to remove extraneous information such as habitat descriptions. Only the relevant locality details were included in the dataset. When specific locality information was essential, it was explicitly detailed in the text or table (Table 1). When necessary, points of occurrence were inferred, based on locations and verified using Google Earth (2024). These inferences were made in specific cases, particularly when only type material was available or when the coordinates of the material were relevant for discussions to be visualised on the map (e.g. *Pitcairniaanomala*, *P.corallina*, *P.geyskesii*, *P.kirkbridei* and *P.sastrei*). Distribution outlier records ("taxonomic suspicious") and specimens with coordinates matching the centroid of municipalities were considered inaccurate and deleted. Our final dataset contains 211 occurrence points (Carvalho 2024), which were filtered from 721 initially acquired points. The maps were created using QGIS (Quantum GIS Development Team 2023) by plotting the collection localities on a Neotropical base map from Hijmans et al. (2004) and the Amazon Rainforest base map from IBGE (2004).

Information about endemism, substrate, vegetation type and occurrence in federative units was taken from Flora e Funga do Brasil (Saraiva et al. 2024) and confirmed, based on the material examined. For species that are not endemic to Brazil, the occurrence information follows Tropicos.org (Missouri Botanical Garden 2024).

## Geographic coverage

### Description

The geographic coverage encompasses eight States in the Brazilian Amazon, including Acre, Amapá, Amazonas, Mato Grosso, Pará, Rondônia, Roraima and Tocantins (Fig. 2).

## Taxonomic coverage

### Description

**Figure 1.**
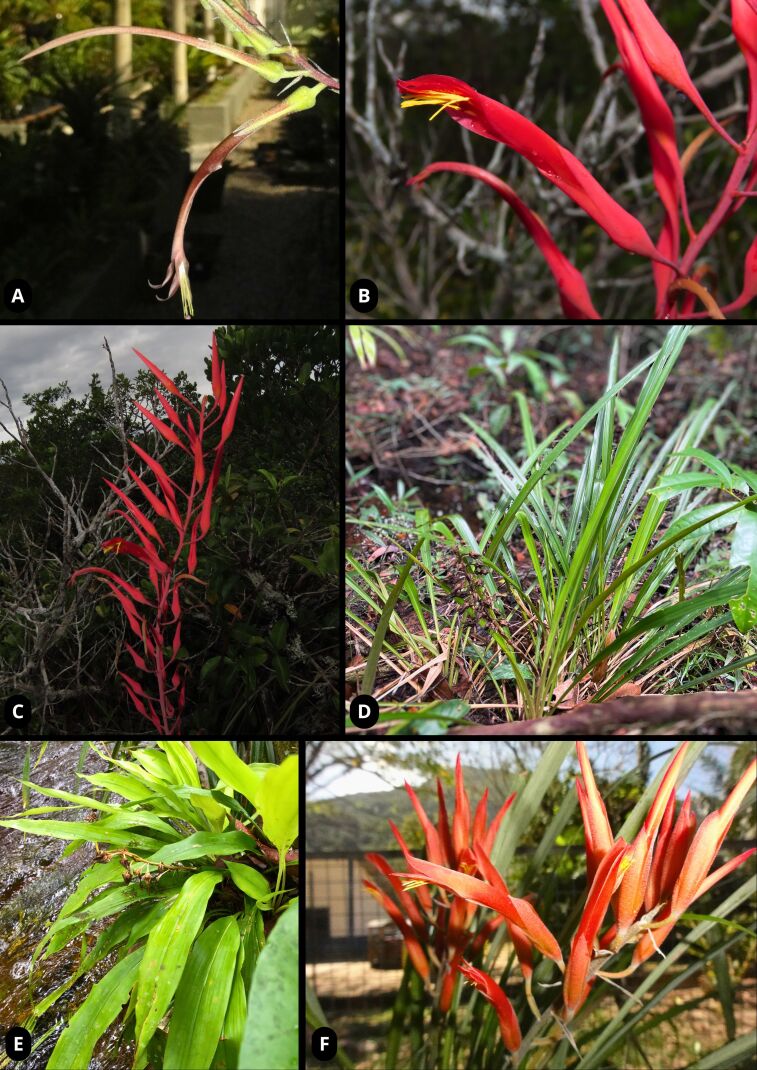
*Pitcairnia* species that inhabit the Brazilian Amazon. **a** *P.burchelli* Mez; **b-c** *P.patentiflora* L.B.Sm.; **d** *P.rubiginosa* Baker; **e** *P.sprucei* Baker; **f** *P.uaupensis* Baker. Photos by B.M. Carvalho (d, e), D.P. Saraiva (a, f) and T. Bochorny & G. Marcusso (b, c).

We recorded the occurrence of 24 species of *Pitcairnia* in the Brazilian Amazon, which are the following: *P.anomala* Hoehne, *P.aureobrunnea* Rauh, *P.burchellii* Mez, *P.caricifolia* Mart. *ex* Schult. & Schult.f., *P.corallina* Linden & André, *P.crinita* E.Pereira & Martinelli, *P.frequens* L.B.Sm. & B.Holst *ex* Saraiva & Forzza, *P.geyskesii* L.B.Sm., *P.kirkbridei* L.B.Sm. & Read, *P.leprieurii* Baker, *P.maidifolia* (C.Morren) Decne. *ex* Planch., *P.neeana* (L.B.Sm. *ex* H.Luther) J.R.Grant, *P.patentiflora* L.B.Sm., *P.poeppigiana* Mez, *P.pulverulenta* Ruiz & Pav., *P.rondonicola* L.B.Sm. & Read, *P.rubiginosa* Baker, *P.sastrei* L.B.Sm. & Read, *P.semijuncta* Baker, *P.sprucei* Baker, *P.torresiana* L.B.Sm., *P.uaupensis* Baker, *Pitcairnia* sp. nov. 1 and *Pitcairnia* sp. nov. 2 (Fig. 1; Table 1).

**Figure 2.**
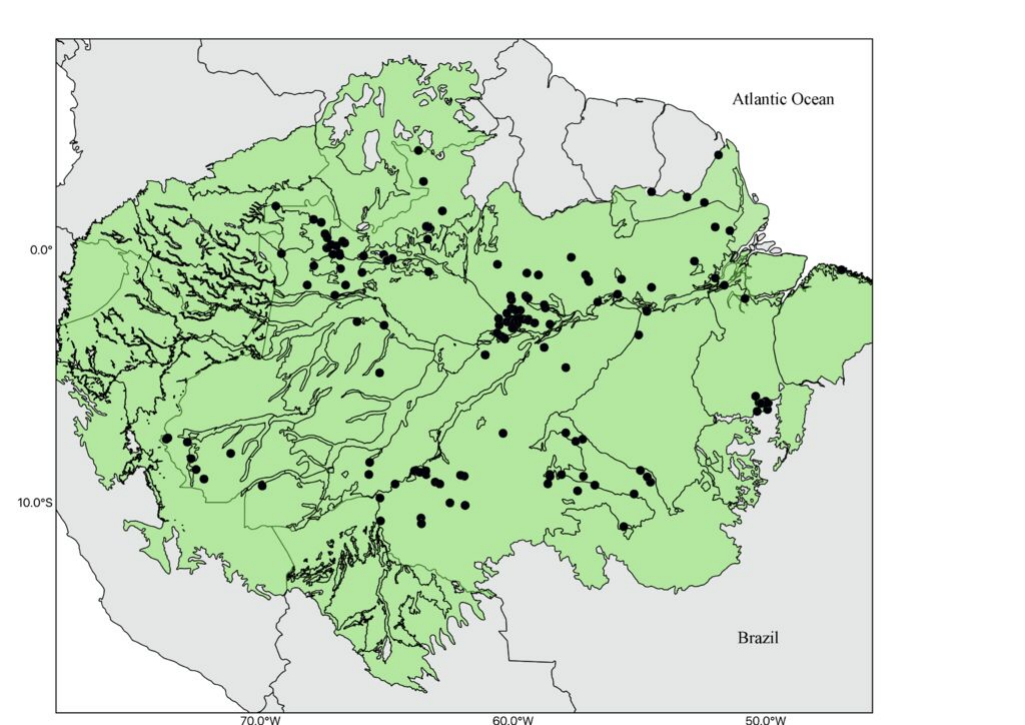
Overview of geographical distribution of *Pitcairnia* (Bromeliaceae) in the Brazilian Amazon.

The Bromeliaceae monograph by Smith and Downs (1974) identifies 13 species of *Pitcairnia* in the Brazilian Amazon. However, the Flora e Funga do Brasil by Saraiva et al. (2024) reports a significantly higher number for the region (23 spp.). Based on our analysis, we confirmed the occurrence of the species listed by Flora e Funga do Brasil (Saraiva et al. 2024), one species was synonymised, one species was recorded for the first time for the Brazilian Amazon (*P.aureobrunnea*) and two new species were discovered and are in the process of being described. To date, this is the highest number of species recorded for the genus in the Amazon Region of Brazil (Fig. 2).

Amongst these species, nine are endemic to Brazil, of which seven exclusively occur in the Amazon domain (*P.anomala*, *P.crinita*, *P.frequens*, *P.neeana*, *P.rondonicola*, *Pitcairnia* sp. 1 and *Pitcairnia* sp. 2) and two (*P.burchellii* and *P.torresiana*) have a stronger association with the Cerrado domain (Table 1). *Pitcairniaburchellii* stands out as one of the species in the genus with the widest distribution in Brazil, where it occurs in states in the Northern, North-eastern, Central-Western and South-eastern regions (Saraiva et al. 2024).

**Figure 3.**
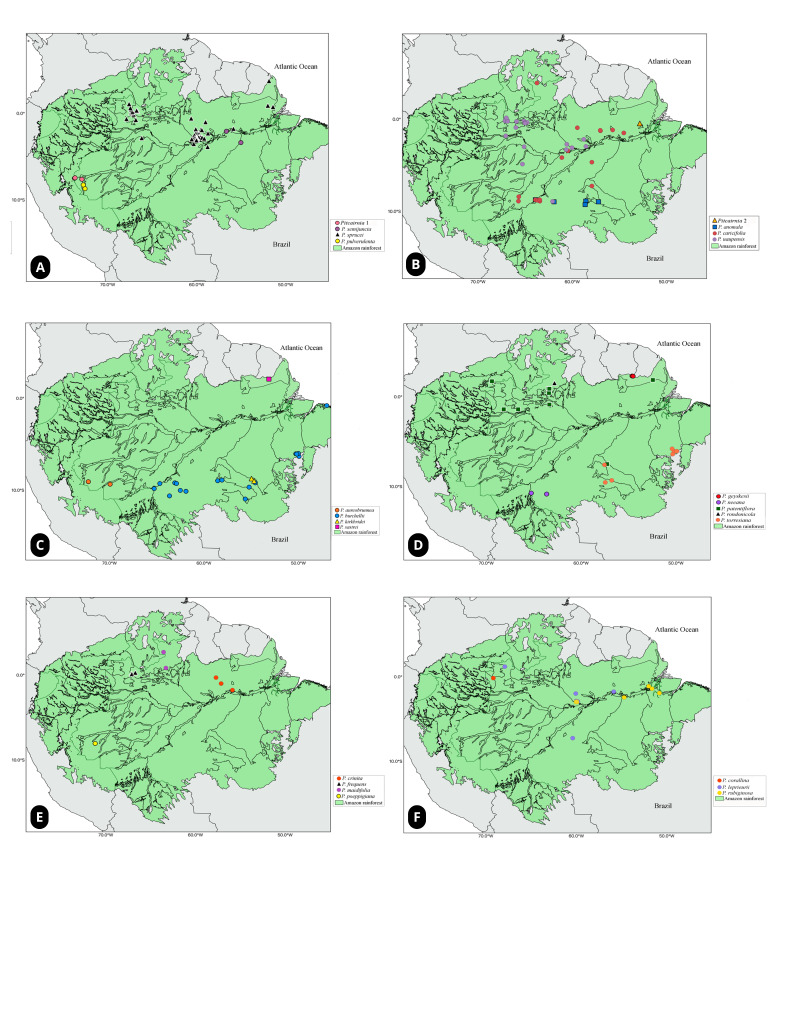
*Pitcairnia* in the Brazilian Amazon: geographical distribution for each species, based on georeferenced herbarium filtered data. **a** *Pitcairnia* sp. 1, *P.semijuncta*, *P.sprucei* and *P.pulverulenta*; **b** *Pitcairnia* sp. 2, *P.anomala*, *P.caricifolia* and *P.uaupensis*; **c** *P.aureobrunnea*, *P.burchellii*, *P.kirkbridei* and *P.sastrei*; **d** *P.geyskesii*, *P.neeana*, *P.patentiflora*, *P.rondonicola* and *P.torresiana*; **e** *P.crinita*, *P.frequens*, *P.maidifolia* and *P.poeppigiana*; **f** *P.corallina*, *P.leprieurii* and *P.rubiginosa*.

The most well-documented species are *P.burchellii*, *P.caricifolia*, *P.patentiflora*, *P.sprucei* and *P.uaupensis*, which are widely distributed throughout the Amazon Basin (Fig. 3: a, b, c and d), occur on a greater diversity of substrates and, in many cases, are in accessible areas, such as anthropogenic zones and along river margins (Table 1). Conversely, many of the species have a low number of records and, in some cases, are only represented by a single collection (*P.rondonicola* and *Pitcairnia* sp. 2) (Table 1). This may be associated with the issues highlighted by Cardoso et al. (2017) and Hopkins (2019), which concern the difficulty of accessing areas and a lack of investment in basic research in the Amazon Region, resulting in undersampled areas. Furthermore, some of these species have records in areas that currently have high anthropogenic activity, which may have led to habitat loss, especially for those with only one record in the Amazon Basin (Table 1).

Most species can be found as rupicolous (19 spp.); eight are exclusively rupicolous, eight are both rupicolous and terrestrial and three are rupicolous, terrestrial and epiphytic. Five are exclusively terrestrial and none is exclusively epiphytic. The significant influence of rivers on the distribution of these species is evident (Fig. 2), considering that 17 species were recorded in forests associated with rivers and other watercourses (Table 1). Unlike many Amazonian groups, whose distribution patterns are often shaped by collection gaps (Stropp et al. 2020, Gomes‐da‐Silva and Forzza 2021), the unique distribution pattern of these species appears to stem from intrinsic biological and ecological characteristicsis.

This strong association with rivers and watercourses underscores the potential role of these habitats in shaping the distribution and diversification of these species. Similar patterns are observed in the Atlantic Forest and Cerrado, where numerous species exhibit a well-defined distribution pattern that is strongly linked with watercourses (Saraiva et al. 2015). This pattern suggests that the strong association of these species with these environments in the Amazon may result from multiple colonisation events over time, as Saraiva et al. (2015) noted, with *Pitcairnia* repeatedly occupying the Amazon Basin, Cerrado and Atlantic Forest through different evolutionary routes. These repeated colonisations likely drove convergent adaptations to these environments, leading to the formation of distinct geographic groups shaped by the unique ecological characteristics of these regions.

## Temporal coverage

### Notes

**Figure 4.**
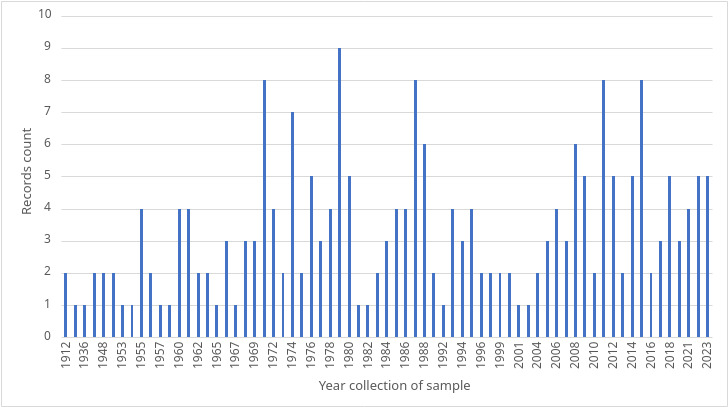
Graph showing the count of *Pitcairnia* records by year.

Our dataset covers collections from 1912 to 2023 (111 years) (Fig. 4) and species published since 1802.

## Usage licence

### Usage licence

Other

### IP rights notes

Creative Commons Attribution License (CC BY 4.0)

## Data resources

### Data package title

Checklist and distribution of *Pitcairnia* species in the Brazilian Amazon

### Resource link

https://doi.org/10.5281/zenodo.14261698

### Number of data sets

2

### Data set 1.

#### Data set name

Checklist of *Pitcairnia* species in the Brazilian Amazon Basin

#### Data format

CSV

#### Character set

UTF-8

#### Description

Checklist of *Pitcairnia* species in the Brazilian Amazon. It contains 24 species that occur in the Amazon Basin. Information on taxonomy, endemism, cccurrence in Brazil, domain, substrate and type of vegetation has been compiled through the study of herbarium collections. The categorisation of vegetation types is derived from Flora e Funga do Brasil and occurrence data for non-endemic species follows Tropicos.org (Missouri Botanical Garden 2024).

**Data set 1.** 

Column label	Column description
ScientificName	The full scientific name with author.
Genus	Name of the genus in which the taxon is classified.
Epithet	Taxon specific epithet.
Author	Author of the monography for the taxon.
taxonRank	The hierarchical level at which the taxon is classified within biological taxonomy, such as species, genus, family etc.
taxonID	A unique identifier assigned to the taxon by the Global Biodiversity Information Facility (GBIF) for organisational and data integration purposes.
taxonRemarks	Additional notes about the taxon, providing context, clarification or details relevant to its identification or status; used to specify if the taxon is a new, undescribed species, if the identification is uncertain or to include any other noteworthy remarks.
GeographicalDistribution	Indicates the countries where the species occurs, using standard codes: BR = Brazil, BO = Bolivia, CO = Colombia; CR = Costa Rica; EC = Ecuador, FR = French Guiana, GT = Guatemala, GY = Guyana, HN = Honduras, NI = Nicaragua, PA = Panama, PE = Peru, SR = Suriname, SV = El Salvador, VE = Venezuela.
OccurrenceBrazil	Brazilian States where taxon occurs: AC = Acre, AL = Alagoas, AM = Amazonas, AP = Amapá, BA = Bahia, GO = Goiás, ES = Espírito Santo, MA = Maranhão, MG = Minas Gerais, MS = Mato Grosso do Sul, MT = Mato Grosso, PA = Pará, RO = Rondônia, RR = Roraima, TO = Tocantins.
Domain	Vegetation Domain where the taxon occurs: Am = Amazônia, Ce = Cerrado.
Substrate	Place where the species occur: Epiphyte, Reophytic, Rupicolous, Terrestrial.
VegetationType	Vegetation type(s) where taxon is present: A = Floresta Ombrófila (= Floresta Pluvial) [Ombrophyllous Forest (Tropical Rainforest)], B = Floresta de Igapó [Inundated Forest], C = Floresta de Terra Firme [Terra Firme Forest], D = Floresta de várzea [Inundated Forest (Várzea)], E = Cerrado (lato sensu), F = Vegetação Sobre Afloramentos Rochosos [Rock Outcrop Vegetation], G = Campo rupestre [Highland Rocky Field], H = Savana Amazônica [Amazonian Savanna], I = Campinarana.

### Data set 2.

#### Data set name

Distribution of *Pitcairnia* species in the Brazilian Amazon

#### Data format

CSV

#### Character set

UTF-8

#### Description

Occurrences of *Pitcairnia* species in the Brazilian Amazon. It contains 211 distribution points for the 24 species present in the Amazon Basin. The dataset was built by compiling data from the studied herbarium collections and contains information on the taxonomy, distribution and other relevant details of the *Pitcairnia* species. This dataset was manually assembled and organised using Microsoft Excel, which was employed solely for data compilation and preliminary analysis.

**Data set 2.** 

Column label	Column description
ScientificName	The full scientific name with author.
Genus	Name of the genus in which the taxon is classified.
Epithet	Taxon specific epithet.
Author	Author of the monography for the taxon.
TaxonRank	The hierarchical level at which the taxon is classified within biological taxonomy, such as species, genus, family etc.
taxonRemarks	Additional notes about the taxon, providing context, clarification or details relevant to its identification or status; used to specify if the taxon is a new, undescribed species, if the identification is uncertain or to include any other noteworthy remarks.
Herbarium	Acronym of the herbarium according to Thiers (2023, continuously updated).
Code	Herbarium voucher code.
Barcode	Herbarium voucher barcode.
RecordedBy	Indicates specimen collector.
RecordNumber	Indicates number collection of sample.
year	Indicates year collection of sample.
month	Indicates month collection of sample.
day	Indicates day collection of sample.
eventDate	Date when the specimen was collected in the format YYYY-MM-DD.
Country	Country where the collection of sample was carried out.
StateProvince	Brazilian State where the collection of sample was carried out.
Municipality	Municipality where the collection of sample was carried out.
Locality	Detailed information about the specific location where the plant specimen was collected.
DecimalLatitude	Record latitude in decimal format.
DecimalLongitude	Record longitude in decimal format.
CoordinateSystem	Coordinate system used: WGS84.

## Additional information

### Conclusions and prospects

The dataset provided here increases what is known about the Brazilian Amazon flora and includes a total of 24 *Pitcairnia* species. In addition to the remarkable diversity, our study also sheds light on the challenges faced by some species, particularly those with few records, which potentially indicates undersampling issues. The distribution patterns of *Pitcairnia* species, notably their preference for rupicolous habitats and environments associated with rivers and other watercourses, underscore the significant impact of these factors on their ecology.

Notably, there is a paucity of collections in the areas adjacent to Brazil's borders with neighbouring countries. The absence of comprehensive data from these regions hinders a holistic understanding of *Pitcairnia* distribution and ecology in the broader Amazon context. This study not only expands our understanding of *Pitcairnia* diversity in the Brazilian Amazon, but also emphasises the urgent need for increased research efforts and conservation initiatives.

Therefore, we hope that these findings provide valuable insights for future studies and conservation strategies aimed at preserving the unique and fragile ecosystems in the Amazon Rainforest. Furthermore, it is heartening to note recent initiatives like Amazonia+10 (CNPq 2023), launched in partnership with CNPq, which promotes calls for scientific expeditions. This initiative represents a positive step in advancing research and conservation efforts in the Amazon, fostering hope for a more sustainable future for this crucial region.

## Figures and Tables

**Table 1.** Information for *Pitcairnia* species in the Brazilian Amazon (occurrence data for non-endemic species follows Tropicos.org (Missouri Botanical Garden 2024)). Distribution, substrate, habitat and the number of records are based on herbarium data, which were personally analysed).

Species	Endemic	Distribution	Substrate	Habitat	Records
*P.anomala* Hoehne	Yes	MT, RO (Juruena and Cururu Rivers)	Reophytic, rupicolous, terrestrial	Forest associated with rivers and watercourses	5
*P.aureobrunnea* Rauh	No (PE)	AC	Rupicolous, terrestrial	Forest associated with rivers and watercourses	2
*P.burchellii* Mez	Yes	PA, RO, TO, AL, BA, MA, GO, MT, MS, MG	Rupicolous, terrestrial	Rocky outcrops; more common in the Cerrado	23
*P.caricifolia* Mart. ex Schult. & Schult.f.	No (BO, CO, VE, FR, GY, SR)	AM, PA, RO, RR	Epiphytic, reophytic, rupicolous, terrestrial	Forest associated with rivers and watercourses	18
*P.corallina* Linden & André	No (CO, PE)	AM	Terrestrial	Forest associated with rivers and watercourses	1
*P.crinita* E.Pereira & Martinelli	Yes	PA (Serra do Cachorro, Oriximiná)	Rupicolous	Forest associated with rivers and watercourses	4
*P.frequens* L.B.Sm. & B.Holst *ex* Saraiva & Forzza	Yes	AM (Morro dos seis lagos, São Gabriel da Cachoeira)	Rupicolous	Mountain rocky outcrops	3
*P.geyskesii* L.B.Sm.	No (FR, GY, SR)	AP (Serra do Tumucumaque)	Rupicolous	Mountain rocky outcrops	2
*P.kirkbridei* L.B.Sm. & Read	Yes	PA (Serra do Cachimbo)	Rupicolous	Forest associated with rivers and watercourses	2
*P.leprieurii* Baker	No (FR, GY)	PA	Rupicolous, terrestrial	Forest associated with rivers and watercourses	5
*P.maidifolia* (C.Morren) Decne. ex Planch.	No (CR, CO, EC, SV, GT, GY, HN, NI, PA, SR, VE)	AM, RR	Rupicolous, terrestrial	Forest associated with rivers and watercourses	2
*P.neeana* (L.B.Sm. ex H.Luther) J.R.Grant	Yes	RO (Guajará Mirim, damaged by intense anthropogenic activity)	Rupicolous	Mountain rocky outcrops	2
*P.patentiflora* L.B.Sm.	No (CO, VE)	AM, PA	Rupicolous, terrestrial	Amazon savannah, mountain rocky outcrops and white sand vegetation	16
*P.poeppigiana* Mez	No (PE, EC)	AC	Terrestrial	Forest associated with rivers and watercourses	1
*P.pulverulenta* Ruiz & Pav.	No (PE)	AC	Rupicolous	Forest associated with rivers and watercourses	2
*P.rondonicola* L.B.Sm. & Read	Yes	AM (Pico Rondon)	Rupicolous	Mountain rocky outcrops	Only type
*P.rubiginosa* Baker	No (CO, VE)	AM, PA	Reophytic rupicolous, terrestrial	Forest associated with rivers and watercourses	12
*P.sastrei* L.B.Sm. & Read	No (FR, GY)	AP (Serra do Tumucumaque)	Rupicolous	Mountain rocky outcrops	1
*P.semijuncta* Baker	No (FR, GY, SR)	PA (Oriximiná)	Rupicolous, terrestrial	Forest associated with rivers and watercourses	1
*P.sprucei* Baker	No (CO, FR, GY, PE, VE)	AM, AP, PA	Epiphytic, reophytic, rupicolous, terrestrial	Forest associated with rivers and watercourses	72
*P.torresiana* L.B.Sm.	Yes	PA, TO, BA, MT, GO	Rupicolous	Mountain rocky outcrops; associated with the Cerrado	9
*P.uaupensis* Baker	No (CO, VE)	AM, RO	Epiphytic, rupicolous, terrestrial	Forest associated with rivers and watercourses	25
*Pitcairnia* sp. 1	Yes	AC (Serra do Moa)	Terrestrial; reophytic	Forest associated with rivers and watercourses	3
*Pitcairnia* sp. 2	Yes	PA	Reophytic, rupicolous	Forest associated with rivers and watercourses	Only type
